# Virtual reality biofeedback interventions for treating anxiety: A systematic review, meta-analysis and future perspective

**DOI:** 10.1007/s00508-021-01991-z

**Published:** 2022-01-06

**Authors:** Oswald D. Kothgassner, Andreas Goreis, Ines Bauda, Amelie Ziegenaus, Lisa M. Glenk, Anna Felnhofer

**Affiliations:** 1 Department of Child and Adolescent Psychiatry, Medical University of Vienna, Vienna, Austria; 2 Department of Clinical and Health Psychology, Faculty of Psychology, University of Vienna, Vienna, Austria; 3 Outpatient Unit for Research, Teaching and Practice, Faculty of Psychology, University of Vienna, Vienna, Austria; 4 Comparative Medicine, the interuniversity Messerli Research Institute, University of Veterinary Medicine Vienna, Medical University Vienna, University of Vienna, Vienna, Austria; 5 Department of Pediatrics and Adolescent Medicine, Medical University of Vienna, Vienna, Austria

**Keywords:** Virtual environment, Psychological treatment, Complementary therapies, Anxiety, Heart rate variability

## Abstract

**Background:**

Virtual reality (VR)-based biofeedback is a relatively new intervention and is increasingly being used for the treatment of anxiety disorders. This is the first research synthesis regarding effects and efficacy of this novel mode of treatment.

**Method:**

We conducted a systematic review and meta-analysis of the VR biofeedback literature on treating anxiety symptoms. The MEDLINE/PubMed, Scopus and Web of Science databases were searched for eligible pre-post comparisons and randomized controlled trials (RCTs). We used self-reported anxiety, heart rate (HR), and heart rate variability (HRV) as primary outcome measures.

**Results:**

A total of 7 studies with 191 participants reported VR biofeedback interventions. Of these studies 5 were RCTs, with 103 participants receiving VR biofeedback and 99 control participants (either 2D biofeedback or waiting list controls). We found that VR biofeedback significantly lowers self-reported anxiety (g = −0.28) and HR (g = −0.45), but not HRV. Furthermore, there were no significant differences in outcomes between VR biofeedback and 2D biofeedback but a significant reduction in HR in the VR biofeedback group compared with the waiting list (g = −0.52).

**Conclusion:**

While the first findings are optimistic, more controlled studies with a wider variety of samples are needed to bring this field forward. Particularly, children and adolescents may profit from the combination of gamification elements, VR, and biofeedback.

## Introduction

Anxiety disorders are among the most common psychiatric disorders with a current global prevalence of 7.3%, ranging from 5.3% in African countries to 10.4% in European/Anglo countries [5]. The development of anxiety disorders is multifactorial. One of the predisposing factors is an increased susceptibility to fear, which can be caused by biological as well as psychosocial factors. Corresponding life events or conditions can trigger an exaggerated fear reaction based on this disposition. Unfavorable coping strategies or reactions of the environment then often lead to escalation or perpetuation of the symptoms [11].

Anxiety disorders are defined as a specific group of diagnoses in the Diagnostic and Statistical Manual of Mental Disorders (5th edition, DSM-5), the most common being specific phobias (e.g., needles, spiders), agoraphobia, social phobia, generalized anxiety disorder, panic disorder and separation anxiety disorder [2]. Symptoms vary widely across these diagnoses with, e.g., fear of losing control, frightening thoughts, poor concentration or hypervigilance for threats. Concomitant physiological symptoms are also common, including for instance, increased heart rate and blood pressure, palpitations, light-headedness, hot flashes, and upset stomach/stomachache. Anxiety disorders often lead to behavioral symptoms, such as avoidance of the (perceived) threat or situations, restlessness, hyperventilation and a need for reassurance [11].

Once anxiety gets triggered corticotropin-releasing hormone activates the locus coeruleus in the brain stem to secrete noradrenaline in order to rapidly activate sympathetic fibers. The sympathetic nervous system then responds immediately by secreting the adrenomedullary catecholamines. Sympathetic activation is reflected in increasing heart rate, blood pressure and respiration [12, 13]. The autonomous nervous system generates the variability between individual heartbeat deceleration and acceleration of consecutive heartbeats. The vagus nerve acts as the operator of the parasympathetic nervous system and predominates at rest. Sympathetic tone increases with increased physical activity or emotional distress. Accounting for both sympathetic and parasympathetic tones, the heart rate (HR) is constantly adapted to challenges of the inner or outer environment. High heart rate variability (HRV) has been associated with an adaptive and healthy cardiovascular system [45], paralleling the overall flexibility of the autonomous nervous system [3]. In contrast, reduced HRV has been linked with autonomic imbalance, represented in various pathologies [47].

The treatment of anxiety disorders consists of different approaches: pharmacotherapy with substances, such as selective serotonin reuptake inhibitors (SSRI) or benzodiazepines for acute alleviation of feelings of tension and fear [48]. Furthermore, psychotherapy and above all cognitive-behavioral therapy as the most effective and most researched therapeutic method [4, 41], is frequently used in anxiety disorders. Another promising and emerging treatment method to alleviate anxiety disorders constitutes biofeedback. Biofeedback refers to a method that integrates the feedback from biosensors, such as electrodes, to make physiological reactions visible to the client in real-time via technical devices. It is an operant training of physiological responding, which stimulates interoceptive self-regulation, which normally is not a conscious process. Regarding the therapy of anxiety disorders, autonomic processes are well-established for feedback via electrodermal activity (EDA), HR and HRV as well as respiratory feedback. Based on the feedback of these signals (e.g., via intonation or visualization), the regulation of physiological processes is usually improved via operant control. Notably, the usage of HRV shows a large significant reduction in self-reported stress and anxiety [23].

A newer form of biofeedback as a therapy for anxiety disorders uses virtual reality (VR) as an addition. Various studies showed a good possibility to simulate distressing stimuli through VR and induce levels of anxiety as well as physiological and subjective stress, which are all similar to a real-life exposure with a comparable physiological stress habituation [27, 30]. Especially the use of virtual exposition therapy shows similar effects compared to traditional in vivo exposition in the case of phobias [10] or in sensu exposition in the case of posttraumatic stress disorder [29]. The mode of functioning in this form of exposition therapy allows an extinction of the fear response but there is also the possibility of stress-buffering through VR, as virtual characters (avatars) can provide effective social support in the encounter of a real stressor [28]. Additionally, VR may be used for implementing game-based approaches for the therapeutic work with children and adolescents [26]. Currently, there are only a few papers that consider the combination of biofeedback and VR affecting anxiety. This paper aims to systematically review existing literature and compare the found papers regarding their effect sizes.

## Methods

### Search strategy and inclusion criteria

**Fig. 1:**
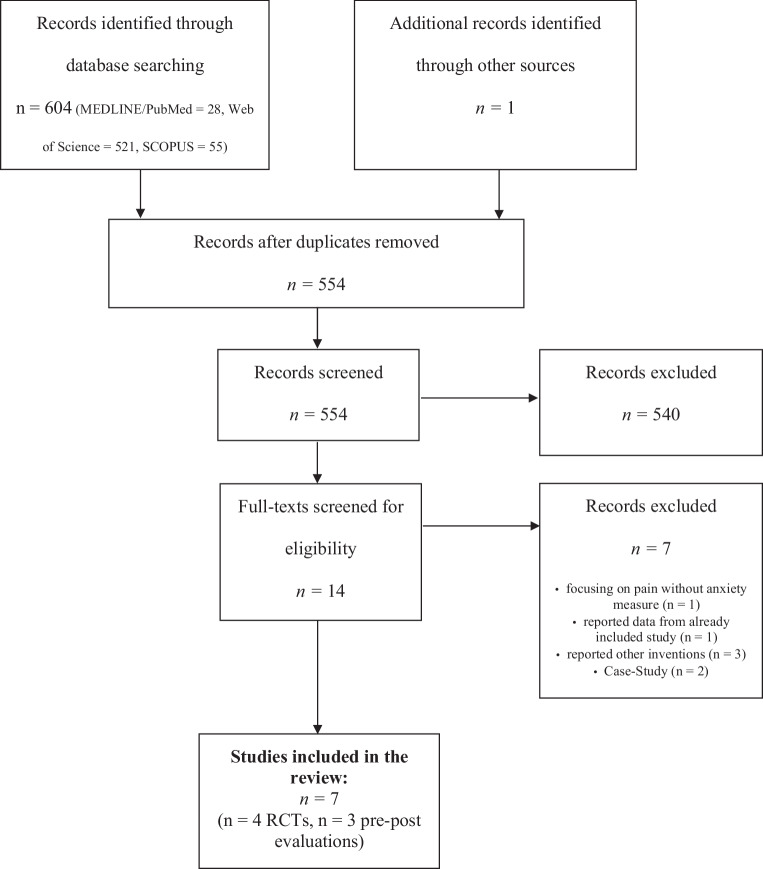
PRISMA chart of screening, exclusion and inclusion criteria

We searched MEDLINE/PubMed, Scopus, and Web of Science using the keywords “Virtual Reality OR VR AND Biofeedback AND anxiety” from the beginning of database records until June 2021. We included studies as eligible for the meta-analysis if they were a randomized controlled trial (RCT) or conducted as a pre-post study, and if they measured effects of (i) anxiety, (ii) HR or (iii) HRV as an indicator of physiological arousal or relaxation. No other inclusion or exclusion criteria were applied. There were no limitations on language or publication status. Furthermore, Google Scholar alerts were enabled to ensure the inclusion of accepted articles and articles in preprint and authors were contacted to ensure the inclusion of unpublished studies. Two authors (ODK and IB) independently examined the title, abstract and the main text of each study and full-text papers were obtained where necessary to evaluate inclusion. Any discrepancies were discussed by the two authors. Final inclusion was based on the following criteria: (i) participants from all ages and genders, (ii) receiving a VR biofeedback intervention, (iii) studies with an active control group (2D-biofeedback) or a pre-post evaluation and (iv) with a focus on the severity of anxiety levels with self-reported anxiety, HR or HRV as outcome variables. Exclusion of documents occurred at each stage (see Fig. 1 for PRISMA flow diagram).

### Data extraction and analysis

First, means and standard deviations of all outcomes were extracted from manuscripts, supplementary material or figures where possible. If means or standard deviations were unavailable they were computed from other summary statistics or statistics of dispersion via the tool Revman (Cochrane Collaboration, London, UK, https://training.cochrane.org/online-learning/core-software-cochrane-reviews/revman). Where unavailable, means and standard deviations were extracted directly from the figures provided in the publications with the software PlotDigitizer (Slashdot Media, San Diego, CA, USA, https://sourceforge.net/projects/plotdigitizer/). To analyze the effect of VR biofeedback (i.e., pre-post comparisons), we computed the standardized mean difference (Hedges’ g) of anxiety symptoms HR and HRV based on means and standard deviations before and after the biofeedback interventions [17]. For HRV, we used the root mean square of successive differences (RMSSD) for HR as a time domain measure used to estimate vagally mediated changes reflected in HRV. We used the formula d = (M_pre_ − M_post_) / SD_pooled_, where M_pre_ is the mean of the measure before the intervention and M_post_ after the intervention, with SD_pooled_ as the standard deviation for both measurements, defined as SD_pooled_ = SQRT(SD_pre_^2^ + SD_post_^2^) / 2 [32]. For the standardized mean difference between intervention and control groups as an indicator of the efficacy of VR biofeedback interventions in RCTs, we calculated Cohen’s d for the post-intervention scores, based on means and standard deviations, with the formula d = (M_Intervention_ − M_Control_) / SD_pooled_, with the respective means of measurements for the intervention and control groups. The calculations of the effect sizes and the subsequent meta-analysis were then conducted using the metafor package for R [51], which automatically corrects Cohen’s d for a potential positive bias in small samples, yielding the effect size Hedges’ g [24]. Following general convention [14], an effect size of 0.20 was considered a small effect, 0.50 a moderate effect and 0.80 a large effect. Random effects models were applied to estimate aggregated effect sizes [8]. All data and codes are stored on a repository of the Open Science Framework (10.17605/OSF.IO/3DHCE).

### Risk of bias assessment

To assess the risk of bias of the included studies, we used predefined criteria based on the AHRQ method guide for comparative effectiveness reviews [52]. Therefore, categories regarding randomization, selection and attrition bias, confounding bias, measurement bias, and statistical problems were applied for coding.

Studies were rated for risk of bias: low risk of bias indicates results can be considered as valid, moderate risk of bias indicates some bias of the study, which probably does not invalidate its results, high risk of bias indicates a significant issue with design, measurement, conduct or analysis, all of which are likely to invalidate the results. Inappropriate or weak methods of randomization, no control for confounding factors, high attrition (≥40%), or differential loss (≥30%), problems in participant selection and moderate to severe statistical problems are predefined indicators for a high risk of bias. The assessments were independently determined by two investigators (IB and AZ); disagreements between the two investigators were discussed and resolved by obtaining a third opinion.

## Results

### Study characteristics

The initial search generated 604 results and after the selection process 7 studies were identified and included in the meta-analysis. The analysis included five studies [6, 7, 42–44] as RCTs. This group of studies covered 103 participants in the VR biofeedback condition and 99 in active control groups using a traditional 2D biofeedback, standard care or waiting lists. All 7 studies were included for pre-post evaluations covering data from 191 participants receiving VR biofeedback interventions; however, one study [50] was designed as a controlled study but reported no active comparator. As Table 1 indicates, 65.9% of participants across the 7 studies were female. One study [49] included children and all others had adult samples. Of the studies three ([6, 43]; [42]) used the State-Trait-Anxiety Inventory (STAI-S/STAI-Y2/STAI-6), one study [50] used the facial anxiety scale (FAS), and one [49] used a self-report state anxiety questionnaire.

**Table 1:** Descriptions of the included studies

Study	Year	Study type	Country	Participants	*n* (TG)	*n* (CG)	% Female	Age M (SD) in years	Comparator in meta-analysis	Groups reported	Main results
Blum et al. [6]	2019	RCT	Germany	Healthy volunteers	31	29	51.7	33.5 (SD = 9.5)	Active control (2D-HRV-Biofeedback)	Standard HRV-biofeedback vs. VR-based HRV-biofeedback	VR-biofeedback increased relaxation self-efficacy and reduced mind wandering
											Participants of the VR-biofeedback group scored higher on the state of mindfulness
											Cardiac coherence and cardiac vagal tone increased in both groups without significant differences
Blum et al. [7]	2020	RCT	Germany	Healthy volunteers	36	–	77.78	21.6 (SD = 4.3)	–	Focused breathing exercise in VR vs. respiratory VR-biofeedback	VR-biofeedback showed significant effects on breathing and focus on the breath
Pallavicini et al. [42]	2009	RCT	Italy	GAD	4	4	75	18–50, M_TG_ = 41.25 (SD = 13.24), M_CG_ = 51.25 (SD = 9.845)	Waiting list	Waiting list vs. VR and mobile phone vs VR and mobile phone plus biofeedback	There was a significant reduction in GAD symptoms and anxiety scores in the VR-Biofeedback group
											Participants gave positive feedback about the introduced mobile phone devices so that they could practice at home
Prabhu et al. [43]	2020	RCT	USA	Preoperative patients with total knee arthroplasty	8	8	75	62.6 (SD = 1.4)	Standard care	Standard care vs. VR-biofeedback	Participants in the VR biofeedback group reported a significant decrease in their perceived anxiety levels preoperative and postoperative
											There was no significant difference in autonomous reactivity between the groups
Rockstroh et al. [44]	2019	RCT	Germany	Healthy volunteers	24	22	60.3	22.9 (SD = 4.0)	a) Waiting list b) active control (2D-HRV-biofeedback)	Waiting list vs. standard HRV-Biofeedback vs. VR-based HRV-Biofeedback	The VR-biofeedback group liked the intervention better than the control group
											They also showed a lower rate of distraction in the VR-biofeedback group
											Concerning the HRV parameters, both biofeedback groups increased most of them; there was a significant decrease in both groups from mid to post
Van Rooij et al. [49]	2016	Pre-Post	The Netherlands	Subclinical anxiety	86	–	39	M = 10.1 (SD = 1.4), 8–12	–	VR-breathing biofeedback	The study found good user experience with satisfying feedback from the participants
											Self-reported state anxiety was reduced in participating children
Venuturupalli et al. [50]	2019	RCT	USA	Rheumatism	10	–	88.24	M = 52.65 (SD = 16.1)	–	Guided meditation following VR-breathing biofeedback vs. VR-breathing biofeedback following guided meditation	A high participant satisfaction with the VR biofeedback was reported
											There was a significant reduction in pain scores (Cohen’s d = 0.50), but no reduction in anxiety scales after the respiratory biofeedback

*RCT* Randomized Controlled Trial, *Pre-Post* Pre-Post-Evaluation Study, *TG* Treatment group, *CG* Control group, *VR* Virtual Reality, *HRV* Heart Rate Variability, *GAD* General Anxiety Disorder

### Effects of VR-biofeedback interventions on anxiety symptoms, heart rate and heart rate variability (pre-post comparisons)

**Fig. 2:**
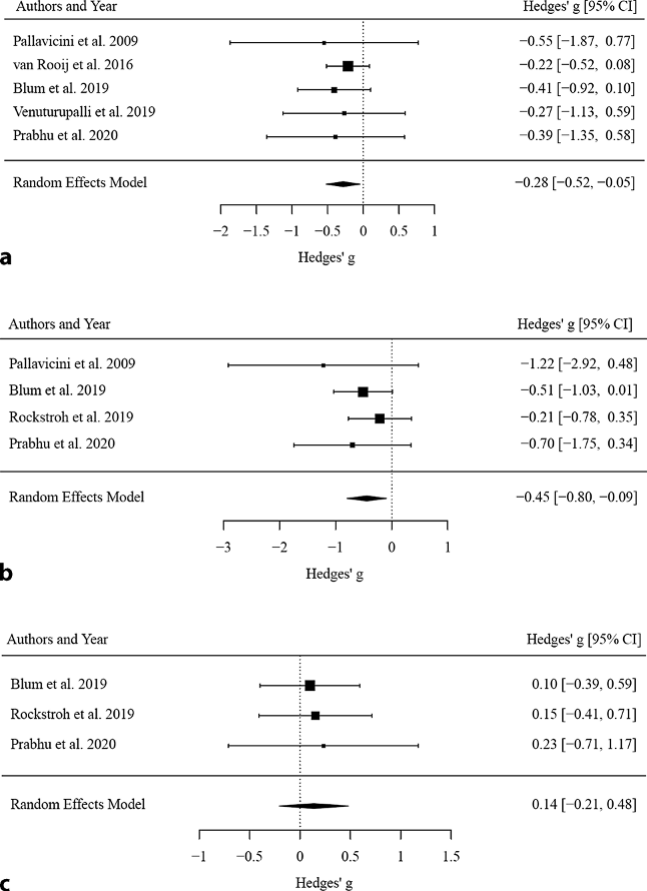
Forest plots of the standardized mean difference (Hedges’ g) of the effect of VR biofeedback on **a** self-reported anxiety, **b** heart rate and **c** RMSSD (pre-post changes)

For anxiety symptoms, k = 5 studies were included in pre-post analyses. The observed outcomes ranged from g = −0.55 to −0.22. The estimated average outcome based on the random-effects model was g = −0.28 (95% confidence interval [95% CI]: −0.52 to −0.05), indicating that the interventions significantly reduced anxiety symptoms (*p* = 0.019). According to the Q‑test, there was no significant amount of heterogeneity in the true outcomes (Q(4) = 0.62, *p* = 0.961, I^2^ = 0.00%). For HR, k = 4 studies were included in pre-post analyses. The observed outcomes for HR ranged from −1.22 to −0.21, and the estimated average outcome based on the random-effects model was g = −0.45 (95% CI: −0.80 to −0.09). Therefore, the average outcome differed significantly from zero (*p* = 0.013). Finally, for RMSSD, k = 3 studies were included in pre-post analyses. The observed outcomes ranged from 0.10 to 0.23, with all of them being positive, i.e., raising the RMSSD score. The estimated average outcome based on the random effects model was g = 0.14 (95% CI: −0.21 to 0.48). Therefore, the average outcome did not differ significantly from zero (*p* = 0.432). All results are displayed in Fig. 2a–c.

### Efficacy of VR biofeedback interventions in randomized controlled trials

For the effect of VR biofeedback interventions on anxiety symptoms in RCTs with waiting list controls, k = 2 trials were included. Both trials reported negative standardized mean differences; however, the estimated average standardized mean difference based on the random effects model was g = −0.56 (95% CI: −1.38 to 0.25). Therefore, the average outcome did not differ significantly from zero (*p* = 0.180). We found that VR biofeedback interventions do not have a greater effect on symptoms of anxiety than waiting list control conditions. Only one study [6] compared VR biofeedback interventions to active controls. The authors reported an insignificant effect of g = −0.01 (95% CI: −0.51 to 0.50, *p* = 0.984).

For the outcome HR compared to waiting list control conditions, k = 3 RCTs were included. The observed standardized mean differences ranged from g = −0.93 to −0.35, with 100% of the estimates being negative. The estimated average standardized mean difference based on the random effects model was *g* = −0.52 (95% CI: −1.00 to −0.05). Therefore, the average outcome differed significantly from zero (*p* = 0.031), indicating that VR biofeedback interventions had a greater effect on reducing HR than waiting list control conditions. For HR compared to active control conditions, no significant differences of the effect were found in k = 2 studies (g = −0.02, 95% CI: −0.40 to 0.36, *p* = 0.909).

For the outcome RMSSD compared to waiting list control conditions, k = 3 RCTs were analyzed. The observed standardized mean differences ranged from −0.17 to 0.55, with the majority of estimates being positive (67%). The estimated average standardized mean difference based on the random effects model was g = 0.31 (95% CI: −0.06 to 0.68). Therefore, the average outcome did not differ significantly from zero (*p* = 0.104), indicating that VR interventions did not have an effect on RMSSD when compared to waiting list controls. Similarly, no effect was found in k = 2 studies with active control conditions, where the difference was g = 0.10 (95% CI: −0.06 to 0.68, *p* = 0.624).

**Fig. 3:**
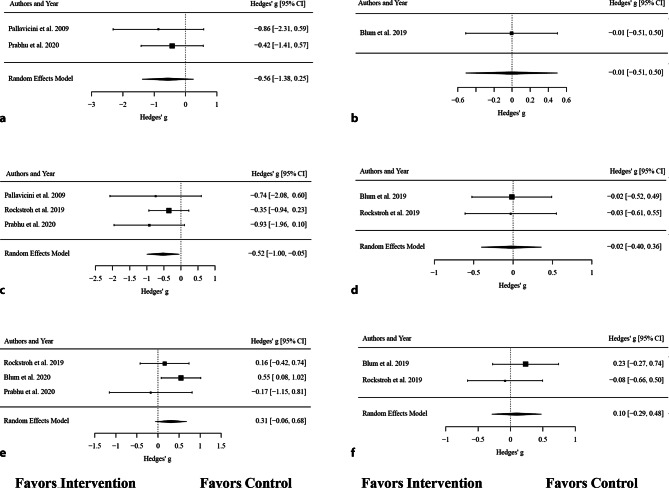
Forest plots of the standardized mean difference (Hedges’ g) of the efficacy of VR biofeedback on **a,** **b** self-reported anxiety, **c,** **d** heart rate and **e,** **f** RMSSD grouped by type of control groups. **a** Self-reported anxiety—Waiting list controls, **b** Self-reported anxiety—Active controls, **c** Heart rate—Waiting list controls, **d** Heart rate—Active controls, **e** RMSSD—Waiting list controls, **f** RMSSD—Active controls

All results are displayed in Fig. 3a–f.

### Risk of bias assessment

**Fig. 4:**
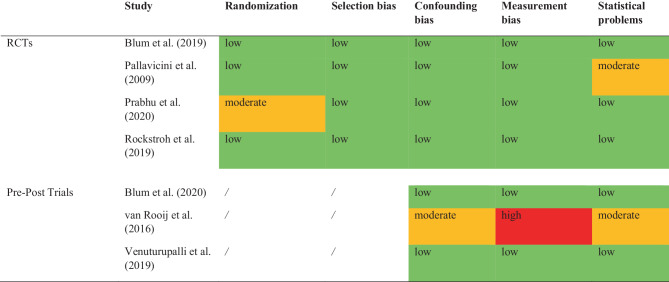
Assessment of quality for low (*green*), moderate (*orange*) or high (*red*) risk of bias based on the AHRQ method guide for comparative effectiveness reviews [52]

This review revealed that the majority of studies showed a low or moderate risk of bias, as presented in Fig. 4. Of the studies four showed an overall low risk of bias [6, 7, 44, 50] and two studies showed a low-moderate risk of bias, predominantly caused by missing information [42, 49].

## Discussion

Given the promising results of using VR [10, 29] to improve the treatment of anxiety disorders, the current meta-analysis set out to systematically review and quantitatively synthesize the effect of a VR enhancement for the biofeedback treatment of anxiety disorders; however, only a small number of studies focusing on VR biofeedback in the context of the treatment of anxiety were found. We included 7 studies with an overall sample of 191 participants receiving a VR biofeedback intervention. Of these studies 5 were randomized controlled trials covering 202 participants in total. Self-reported anxiety, HR, and RMSSD were used as outcome variables. We found a significant effect with a small effect size (g = −0.28) regarding the effect of VR biofeedback interventions in pre-post comparisons, which indicates that the VR biofeedback lowers experienced anxiety levels. Moreover, pre-post comparisons indicated a significant reduction in HR with moderate effect size (g = −0.45) but no effect on RMSSD. Analyzing the aggregated effects in RCTs with waiting list controls, there was no effect between the groups regarding self-reported anxiety and RMSSD, but a significant reduction in HR with moderate effect size (g = −0.52). Additionally, no differences were found in comparison with active controls (2D biofeedback). These results suggest that VR biofeedback could be a useful tool in the treatment of anxiety disorders but more studies are needed to strengthen these preliminary findings.

### Effects of virtual reality and biofeedback

For traditional biofeedback, several obstacles have been described which may limit both its usability and effectiveness for treating anxiety disorders. Among these, keeping up the patient’s motivation and engagement have repeatedly been discussed as an issue [22, 53]: stimuli used in classical biofeedback treatment have been regarded as quite abstract, complex, or not appealing to patients, hence, leading to low intrinsic motivational levels and the difficulty of keeping up training motivation over several sessions. Furthermore, keeping up focused attention during the training sessions has been identified as another challenge in biofeedback training [6]. While this may be connected to the issue of unappealing or abstract task content, problems of sustaining attention may also be related to distractions from the environment and one’s own inability to immerse oneself into the task.

For these challenges, VR may constitute an optimal solution. Technological developments enable the programming of appealing content depicting either real or phantasy surroundings. Repeatedly, VR has been shown to successfully induce anxiety levels, both subjectively experienced and physiological, which are comparable to those observed under real-life conditions [27]. Furthermore, the inclusion of gamification elements [15] in the virtual worlds promises to exert positive effects on motivation through the implementation of incentives as well as a narrative and game progression. Particularly in children and adolescents, the use of gamification elements is thought to positively impact involvement and motivation [18]. Furthermore, using immersive hardware, such as VR glasses, which cover the entire field of view, is likely to minimize the influence of environmental distractions and increase attentional focus on the task or stimulus itself [46].

### Feasibility and safety data

Overall, this review adds more sustenance to the assumption that combining VR and biofeedback provides advantageous feasibility and user experience. Results indicate that VR biofeedback is feasible and that it leads to high levels of satisfaction not only in children and adults with anxiety disorders (children: [49]; adults: [44]) but also in patients treated for pain [50] and in the context of surgery (preoperative and postoperative [43]). Future research can thus expand its focus to go beyond traditional therapeutic areas of application and test for the usability, applicability, and acceptability of VR biofeedback also in other patient groups and contexts.

A promising approach seems to lie in the use of mobile systems particularly: A study implies that patients see it as beneficial using biofeedback at home via mobile devices [42]. Currently, a broad range of mobile phone apps exist, which enable assessment of several physiological parameters (e.g., HR, skin temperature) via internal or external sensors and may also be used to communicate with healthcare professionals (Weerdmeester et al. 2020). Furthermore, these APPs may be combined with gamification elements to enhance engagement and motivation. Overall, making use of smartphone APPs and so-called wearables (e.g., smartwatches) may all expand the scope of traditional biofeedback also to include hard to reach patients, such as those residing in rural areas or those who are bound to their homes because of disability or chronic illness.

While VR in all forms, be it in immersive VR glasses or as a smartwatch APP, is a promising tool for biofeedback, less is known about its safety and possible adverse effects. Most studies included in this review and meta-analysis, did not provide any information on adverse effects or safety issues. Only one study [50] reported having excluded three patients because of motion sickness. These patients showed symptoms of active nausea or vomiting and had a history of chronic vertigo or dizziness. In general, these issues are underreported and future studies need to actively consider assessing possible contraindications and adverse effects of VR biofeedback.

### Therapeutic advantages of virtual reality biofeedback

Several studies showed promising effects with respect to reducing anxiety levels and symptoms related to anxiety disorders [42, 49] as well as regarding vagal tone when compared to 2D biofeedback [6]. Another positive effect seems to lie in lower distraction levels and less mind wandering during VR biofeedback when compared to 2D biofeedback [6, 44]. Similarly, the attentional focus was increased for respiratory tasks in VR [7]. Yet, it is too early to draw conclusions with respect to the stability of these effects, as most studies included in this review and meta-analysis only examined a single session.

Additionally, it remains unclear why the positive effects of VR biofeedback were limited to the subjective experience of anxiety and to HR. In contrast, RMSSD did not show any changes in respective studies; however, a recent review has highlighted the vital role of HRV on emotional well-being. Higher levels of HRV were not only correlated with higher emotional satisfaction but also with lower levels of anxiety, worry, and rumination [37]. With respect to health promotion, resonance breathing exercises in biofeedback interventions may increase respiratory efficiency by making more blood available during inhalation when the concentration of oxygen in the alveoli of the lungs is at a maximum [55]. In comparison to spontaneous breathing, prolonged expiration during an incremental exercise leads to more effective ventilation, increased parasympathetic tone and decreased sympathetic nervous system activity [38]. It has been suggested that coherent breathing activates high amplitude oscillations that ultimately affect brain rhythmicity, especially in regions associated with emotion regulation [40].

The efficacy of HRV has several clinical and behavioral implications for individuals suffering from anxiety. Reductions in resting HRV have been previously linked with social interaction anxiety. The higher the HRV reduction was the more severe were the symptoms of social interaction anxiety, fear, and avoidance [1]. Interestingly, in women with more social anxiety symptoms, lower RMSSD scores measured during an emotion recognition task were associated with higher recognition accuracy [36]. In males, cooperative social behavior was facilitated by increased vagal tone [35].

Future studies would be essential to identify which virtual scenarios and biofeedback protocols (e.g., including the target biofeedback indicator, the number, frequency and duration of sessions) might be more effective in raising parasympathetic tone. In addition, it has been demonstrated that amusing stimulation triggers parasympathetic responses [54] and thus VR biofeedback intervention content might be adjusted accordingly.

### Future perspectives for research

There are some current novel interventions based on VR, such as virtual naturalistic developmental behavioral interventions for autism (e.g., [16]), natural environments for relaxation techniques for stress reduction (e.g., [25]), self-guided for specific phobias (e.g., [34]), or automated VR treatments for psychosis (e.g., [20]). A critical element in most of these future developments seems to be the use of specific game design principles in a non-gaming context, such as healthcare (see [39]). Such design principles are closely associated with the mechanics of games and a recent review [21] has, amongst others, identified the following core characteristics: personalization of avatars, adaptive game mechanics, rewards (e.g., points, virtual currency, badges), storyline, immediate feedback and progress status.

Given the assumption that such gamification elements may increase motivation and attentional focus and that the use of immersive VR technologies, such as VR glasses may decrease distractions and increase engagement with content, the combination of VR with biofeedback may be of particular benefit to children and adolescents. More than adults, children and adolescents may struggle with keeping up their motivation and goal orientation over the course of treatment [18]. Hence, this group of patients may especially profit from using VR technology and gamification elements. Interestingly, however, data on VR-based treatment in children and adolescents is still scarce [31]. First results on incorporating gamification elements into classical biofeedback training for children [26] and on using VR biofeedback in children [9, 49] are promising; however, more carefully designed controlled studies are needed, which make use of both gamification and virtual reality for biofeedback therapy.

**Fig. 5:**
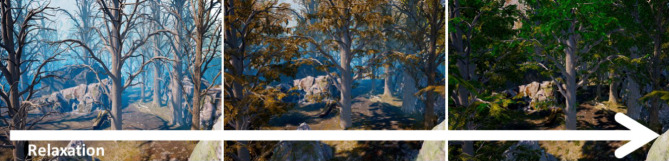
Game-based virtual reality biofeedback for children and adolescents Relaxation scenario (environment translates during induced relaxation: images following from low to high relaxation level) in a game-based virtual reality biofeedback of children and adolescents with anxiety disorders (Conquer Catharsis) [33]. (Copyright and with permission by Andreas Lenz & Helmut Hlavacs)

Currently, an RCT examining a game-based virtual reality biofeedback program in children and adolescents, is underway (see [33]). The program runs via fully immersive virtual reality glasses and depicts a phantasy island environment, which can progressively be explored by participants upon solving one task after the other: these tasks include changing elements of the environment dynamically through reductions of HR levels. As such, children and adolescents are, for instance, required to make leaves grow on trees (see Fig. 5) or to open doors to be able to proceed further and ultimately reach the top of the island mountain.

Despite these upcoming, highly promising developments, it is important to keep in mind that using VR technology requires appropriate training and know-how on the part of healthcare professionals. Also, not all technologies are suitable for medical/therapeutic purposes. Thus, before implementing them in the therapeutic context, the responsible professional must check whether the requirements for safe and autonomous use (e.g., Internet access and the possession of a smartphone in the case of APP-based applications) are met. Also, the use of fully immersive VR technology must be embedded in an evidence-based treatment plan and should always be accompanied by qualified personnel because of its potential to evoke intense emotional responses [19].

Overall, the permeation of smartphones in everyday life offers novel possibilities for treating children and adolescents with biofeedback. Like adults, they may profit immensely from integrating treatment elements into their daily routine (Weerdmeester et al. 2020), particularly if the applications incorporate gamification elements and invite children to play the game repeatedly. This gamification approach might be an effective tool for making technology-mediated treatment more attractive for patients, especially children and adolescents. Furthermore, future trials testing VR biofeedback should focus on longer treatment durations compared with active treatment (e.g., 2D biofeedback) with a sufficient number of participants. This would allow better predictions of the efficacy and effectiveness of this treatment approach. These trials should further corroborate the effects of VR biofeedback to treat anxiety disorders using a multilevel biofeedback consisting of various physiological parameters (e.g., EDA, EMG). It is of importance that patients are not overwhelmed at the beginning of a VR biofeedback; therefore, a distinct initiation phase should be implemented, especially for patients with less VR experience. Additionally, future trials should address longitudinal data regarding the stability of symptoms. Notably, no study included in this review reported long-term follow-up data or examined whether interventions reduced anxiety levels or physiological responses in participants’ daily lives. These subsequent trials should also include ecologically valid assessments to measure changes of symptoms in daily life during and after the treatment period.

## Conclusion

There are only a limited number of studies in this field, and more studies are needed for a proper assessment of efficacy, effectiveness and safety issues. Future trials should cover more sessions for controlled evaluations as well as also include children and adolescents. Nevertheless, this research suggests that virtual reality biofeedback interventions seem to be a promising augmentation of traditional 2D biofeedback for treating anxiety symptoms.
